# Chronic cannabis consumption and physical exercise performance in healthy adults: a systematic review

**DOI:** 10.1186/s42238-020-00037-x

**Published:** 2020-10-07

**Authors:** Andrew Kramer, Justin Sinclair, Lara Sharpe, Jerome Sarris

**Affiliations:** 1 NICM Health Research Institute, Western Sydney University, Locked Bag 1797, Penrith, NSW 2751 Australia; 2 School of Psychology, Western Sydney University, Penrith, Australia; 3 Department of Psychiatry, The Melbourne Clinic, Professorial Unit, The University of Melbourne, Melbourne, Australia

**Keywords:** Cannabinoids, Cannabidiol, Marijuana, Cannabis, Physical activity, Exercise, Sport, Athletics

## Abstract

**Objective:**

The effects of chronic cannabis consumption on physiological parameters of athletic performance are investigated to determine whether chronic cannabis consumption negatively affects athletic performance; improves performance, potentially via enhanced recovery; or has no effect at all.

**Methods:**

A systematic review of the literature (cross-sectional, longitudinal, and intervention studies) concerning the effects of cannabis consumption on sports performance outcomes, e.g. VO2Max (maximal oxygen uptake), PWC (physical work capacity) up to January 2020 was conducted using the PubMed, CINAHL, Medline, PsycArticles, PsycInfo, SPORTDiscus, Psychology and Behavioural Sciences Collection, and Health Source: Nursing/Academic Edition databases. After screening and additional forward searching, four articles were found to fit the inclusion criteria.

**Results:**

Resting heart rate was the only physiological measure that significantly differed between groups, and only in one of the four studies included herein. The strongest predictors of athletic performance (VO2Max and PWC) were not found to be significantly different between groups in any of the included studies. Chronic cannabis consumption had no significant effect on athletic performance. The included studies did not assess other elements, such as recovery or endurance.

**Conclusion:**

No evidence exists for ergogenic or ergolytic effects from chronic cannabis consumption. In some sports, advantages may plausibly be conveyed by psychotropic enhancement or pain reduction. Further research (particularly longitudinal or interventional studies) is required to determine whether cannabis, or constituents thereof, may provide indirect supplemental benefits to athletes.

## Introduction

The establishment of the World Anti-Doping Agency (WADA) and subsequent penalisation of athletes for cannabis use has prompted greater scrutiny of the effects of cannabis effects on athletic performance (Hilderbrand 2011). Docter et al. (2020) reviewed the epidemiology of cannabis use in student and elite athletes, finding that approximately one in four had used cannabis in the past year and that athletes commonly believed that cannabis would negatively affect their performance, consistent with research findings suggesting that cannabis is non-ergogenic and potentially ergolytic.

While Δ9-tetrahydrocannabinol (THC) is considered the main psychoactive constituent, cannabis contains hundreds of potentially biologically active chemicals, some of which may provide synergistic or “entourage” effects, and some revealed less potent psychotropic effects, including cannabidiol (CBD) (Russo 2011). McCartney et al. (2020) examined the potential effects and applications of cannabidiol (CBD) in sports, based on clinical trials, animal models and in vitro studies, finding that further research is needed to determine if CBD conveys analgesic, anti-inflammatory, anxiolytic and neuroprotective benefits; protection against exercise-induced gastrointestinal damage; or enhanced bone fracture healing. Huestis et al. (2011) proposed that anxiolytic, euphoric, and perceptual enhancement effects of cannabinoids may provide advantage in specific sports such as archery and shooting.

Maximal oxygen uptake (VO2Max) is a gold standard measure of cardiorespiratory fitness and a strong predictor of an athlete’s ability to maintain peak performance (Bassett and Howley 2000). Peak work capacity (PWC) or ‘peak power’ estimates the maximum power a person is capable of outputting. In modern studies, peak power is usually measured using the Wingate methodology. Kaminsky et al. (2014) suggests that PWC measures of overall exercise tolerance better, whereas VO2Max is more specific to cardiovascular conditioning. Blood pressure (BP), heart rate (HR) and lung capacity (often measured by one-second Forced Expiratory Volume; FEV1) are secondary measures of interest that detect isolated components of aerobic performance. Elevated BP decreases exercise performance (Mazic et al. 2015), HR is a key factor in cardiac output until intensity nears VO2Max (Munch et al. 2014) and lung capacity restricts oxygen intake.

To determine if scientific grounds exist for regarding cannabis as a potential doping agent, Trinh et al. (2018) performed a systematic review, finding only three studies fitting their criteria, published between 1975 and 1986 (Maksud and Baron 1980; Renaud and Cormier 1986; Steadward and Singh 1975). These studies show a small ergogenic effect on FEV1 via bronchodilation, and an opposing ergolytic effect on anaerobic performance, as measured by PWC. The bronchodilation finding is consistent with Tashkin et al. (1975), in which bronchospasm was induced in asthmatic patients via methacholine inhalation and via exercise on separate occasions. Participants were given either a 2% THC joint or placebo once bronchospasm was achieved, those receiving THC rapidly recovered in both conditions. Kennedy (2017) suggests that while THC could benefit asthmatics, common asthma medications are more effective and demonstrate fewer side effects.

Comparing older studies presents difficulties, as PWC measurement methodologies were inconsistent, using different workloads, increments and intervals. VO2 measures are more comparable, as the gas analysis is less dependent on the exertion task. Past design and reporting standards present further challenges, small samples were common and the THC content of cannabis used was typically around 2%, much lower than commonly imbibed varieties. Avakian et al. (1979) compared the exercise performance of six chronic cannabis users without cannabis, after cannabis (1.54% THC) consumption, and after smoking a placebo. No significant effects were found on VO2, or physiological measures, except heart rate, which was higher during rest, exercise and recovery phases in the cannabis condition. Renaud and Cormier (1986) compared healthy adults with and without cannabis (1.7% THC) in a crossover trial, observing slightly greater VO2 during the cannabis condition at 60–80% of maximum exertion, but no significant difference at maximum exertion. Decreased Physical Work Capacity was observed in the cannabis condition, likely the result of prematurely achieving maximum HR due to cannabis induced tachycardia.

Acute and chronic effects of cannabis consumption differ substantially. Acute use of cannabis in non-users induces tachycardia, however, as shown by Benowitz and Jones (1975), this effect rapidly fades with regular consumption of cannabis. Benowitz and Jones also observed decreases in resting HR and BP, and decreased BP elevation in response to exercise. These effects increased with dosage, and during the early maximal dosing phase were so pronounced that two of the twelve male participants were unable to complete exercise tasks due to dizziness. Hollister et al. (1968) acutely administered either THC or a synthetic analogue in men aged 21 to 44 and observed similar drops in BP, elevated HR, and impaired strength (measured on a finger ergograph). Hollister et al. found that dizziness was common, and two of the 29 participants experienced syncope when attempting to stand. In addition to tachycardia and hypotension, Goyal et al. (2017) describe an association between cannabis use and acute cardiovascular events including arrhythmias, arteritis and myocardial infarctions. Goyal et al. report an escalated risk when cannabis use is combined with regular cigarette use and/or intense exercise. Kennedy (2017) systematically reviewed past research on the relationship between cannabis and exercise, including those within patient populations, noting that in angina patients, exercise-induced angina occurs more quickly due to cannabis-induced tachycardia.

Gillman et al. (2015) comprehensively reviewed the literature on cannabis and exercise, including interactions with the endocannabinoid system, noting the absence of studies identifying the psychological impacts of cannabis on athletic performance. YorkWilliams et al. (2019) sought to resolve that lack by surveying adults in “legal” states regarding the use of cannabis with exercise. The most endorsed statements were that enjoyment of exercise and recovery from exercise were enhanced. Substantially fewer endorsements were seen for performance and motivation. Lisano et al. (2019a) also surveyed cannabis users about exercise-related use, finding the most reported reason was pain management. In contrast to YorkWilliams et al., 77% of Lisano, Phillips, et al.’s sample felt that their performance was enhanced by cannabis, with many respondents suggesting focus or “flow” effects as their reason for using before or during exercise. Zeiger et al. (2019) surveyed athletes ranging from recreational to elite and found an overwhelming majority of participants reported calming, pain reduction, and sleep aid effects from cannabis. Gillman et al. recommend exploration of the endocannabinoid (eCB) system, which is likely linked to the “runner’s high” experienced by many athletes, previously attributed to endorphins (endogenous opioids). The eCB system is proposed to affect exercise motivation through activation of dopaminergic reward pathways and may be subject to modulation by exogenous cannabinoids, however, it is unclear whether such modulation would enhance or diminish endogenous effects, or if motivational effects differ between acute and chronic use.

This systematic review aims to complement existing reviews, which primarily report on acute effects of cannabis use, by reviewing the available data on (1) the effects chronic cannabis use has on fitness measures; (2) any effects chronic cannabis use has on physical activity levels; (3) what effect chronic cannabis use has on actual sport performance.

## Methods

An initial scoping search of the literature was conducted to determine the viability of the area for analysis. Several review articles were found, suggesting sufficient literature existed within this area.

A search of the PubMed, CINAHL, Medline, PsycArticles, PsycInfo, SPORTDiscus, Psychology and Behavioural Sciences Collection, and Health Source: Nursing/Academic Edition databases was conducted, accessing articles up to January 2020 using the following search terms:

“Cannabi*” OR “marijuana” OR “marihuana” OR “THC” OR “tetrahydrocannabinol” OR “delta-9-tetrahydrocannabinol” OR “CBD” AND “Athlet*” OR “physical activity” OR “fitness” OR “exercise” OR “sport” OR “endurance” OR “VO2” OR “VO2Max”.

The search and data abstraction were performed by AK, studies with potential relevance were reviewed by the other authors for inclusion. Studies were included if they: reported validated measures of athletic performance, physical activity level, or physiological parameters, or a quantifiable individual measure of performance in a sport; involved healthy humans aged 16–60 who use cannabis recreationally; were cross-sectional or longitudinal, or prospective interventional clinical trials comparing cannabis to a placebo. Studies were excluded that focused on acute effects of cannabis, reported on populations other than physically and mentally well adult humans, or had reduced cannabis use as an outcome measure. The age of studies and gender of participants were not restricted; however, the literature review was confined to English language reports only.

**Fig. 1:**
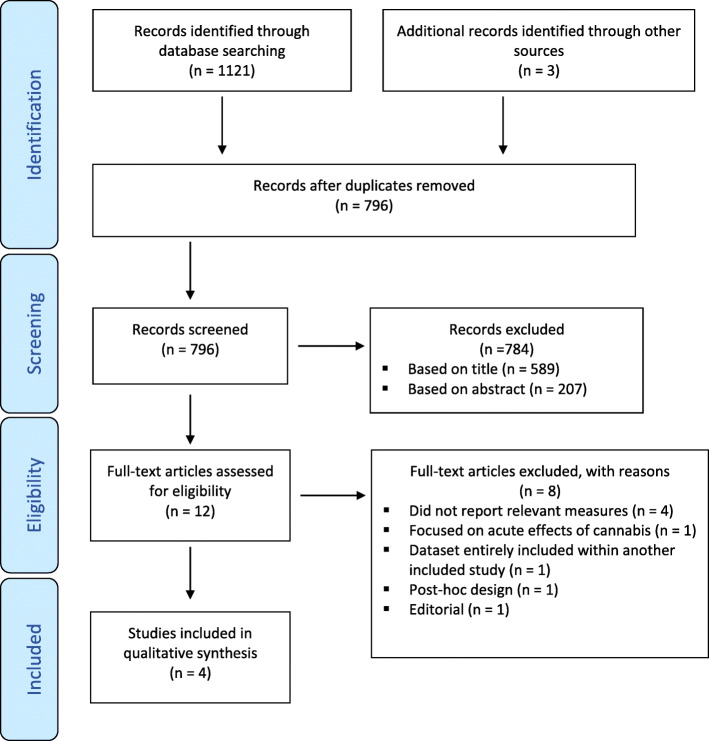
PRISMA flowchart of systematic literature search . n = number of records, PRISMA = Preferred Reporting Items for Systematic Reviews and Meta-Analyses (Moher et al. 2009)

## Results

### Studies data overview and synthesis

A total of 1121 articles were identified, with an additional three articles found by forward searching of relevant reviews and articles earmarked for potential inclusion (Fig. 1). Of those, 328 were duplicates, leaving 796 articles, of which 12 were determined to require full-text evaluation. Five articles were determined to meet the inclusion criteria, however, Wade et al. (2019a) was excluded due to the study population being entirely included within Wade et al. (2019b). The common characteristics of these studies were the inclusion of generally healthy males and females aged between 16 and 39, the use of cross-sectional designs to compare cannabis users to non-users, and the use of physiological measurements of cardiorespiratory performance. Lisano et al. (2019b) and Lisano et al. (2020) recruited participants from a university campus, Wade, Gilbart, et al. used local newspaper advertisements, and Maksud and Baron (1980) recruited through local workers unions. Lisano, Kisiolek, et al. and Lisano, Smith, et al. defined chronic users as those using cannabis at least once per week for the past 6 months, Wade, Gilbart, et al. similarly defined them as using cannabis more than 52 times in the past year, whereas Maksud and Baron classified cannabis users as using several times per week. All included studies allocated groups based on self-reported cannabis use. Lisano, Smith, et al. confirmed the presence of cannabis metabolites in most of their users and absence of metabolites in all non-users. Mean cannabis usage within user groups was reported in three studies. Lisano, Kisiolek, et al. reported mean usage of 18 days in the last 30, with 1.67 uses per day used. Lisano, Smith, et al. reported mean usage of 21 days in the last 30, with 1.79 uses per day used. Wade, Gilbart, et al. reported a mean past-year usage of 422 joints. Lisano, Kisiolek, et al. and Lisano, Smith, et al. required participants to meet WHO physical activity recommendations and obtained samples with mean VO2Max scores of 50.31 and 52.12 respectively, Maksud and Baron recruited labourers whose mean VO2Max was 38.22, Wade, Gilbart, et al. did not restrict fitness levels, but did restrict age to 16–26 and obtained a mean VO2Max of 42.64, suggesting substantially lower fitness levels than the Lisano samples (see Table 1).

**Table 1:** Fitness measures used in studies

Study authors	Relative VO2Max *M* (SD)		*p*	Cohen’s *d*	Peak Work Capacity *M* (SD)		*p*	Cohen’s *d*
	Non-users	Cannabis Users			Non-users	Cannabis Users		
Lisano et al. (2020)	52.43 (5.79)	48.18 (8.37)	.12	.60				
Lisano et al. (2019b)	53.16 (5.26)	51.08 (8.88)	.49	.28	871.52 (150.7)	920.55 (185.83)	.49	.29
Maksud and Baron (1980)	37.15 (5.65)	39.40 (7.61)			200.25 (46.09)	197 (38.22)		
Wade et al. (2019b)	41.52 (10.65)	43.98 (9.05)	.27					

*VO2Max* Maximal oxygen uptake, *M* Mean, *SD* Standard Deviation, *p* Probability of attaining results if null hypothesis is true, *Cohen’s d* Effect size. Values left blank where not provided

Relevant outcomes from among the selected studies were VO2Max, PWC, other pulmonary measures, strength and endurance measures (grip strength, side plank time, hip flexion torque, etc.), perceived exertion, resting heart rate, and blood pressure (See Tables 2 and 3).

**Table 2:** Summary of included studies

Study	Design	Sample size	Participants	Intervention	Outcomes
Lisano et al. (2019b)	Cross-sectional observational study design comparing cannabis users (*n* = 12) and non-users (n = 12)	24	Physically active males aged 19–39 (M = 23.71)	N/A	Vo2Max, Physical Work Capacity, pulmonary function, perceived exertion, BP, BMI, Body fat%, strength, serum testosterone, cortisol
Lisano et al. (2020)	Cross-sectional observational study design comparing cannabis users (n = 15) and non-users (*n* = 15)	30	Physically active male (n = 10) and female (*n* = 5) cannabis users and physically active male (*n* = 10) and female (n = 5) non-users	N/A	VO2Max (treadmill), HR, BP, BMI, body fat%, serum cortisol and inflammatory markers
Maksud and Baron (1980)	Cross-sectional observational design using four groups: Cannabis and cigarette users (*n* = 18), cannabis only (*n* = 13), cigarette only (*n* = 17), non-user (n = 17)	65	Male blue collar workers aged 19–34	N/A	VO2Max, Physical Work Capacity, body fat %, lean body weight, hematocrit (%), hemoglobin, HR, perceived effort
Wade et al. (2019b)	Cross-sectional observational design comparing cannabis users (38) and non-users (45)	83	English speaking persons aged 16–26, including 24 female and 21 male non-users, 12 female and 26 male cannabis users	N/A	VO2Max, average physical activity, BMI, psychological measures

*HR* Heart Rate, *VO2* Volume Oxygen, *BMI* Body Mass Index, *BP* Blood Pressure, *N/A* Not Applicable, *n* Number of participants in a group

**Table 3:** Outcomes used in studies

Study Authors	Outcomes	Intervention Between Groups	# of participants (studies)	
			Cannabis Users	Non-Users
	VO2Max	NS	96 (4)	106 (4)
Lisano et al. (2019b)				
Lisano et al. (2020)				
Maksud and Baron (1980)				
Wade et al. (2019b)				
	PWC	NS	43 (2)	46 (2)
Lisano et al. (2019b)				
Maksud and Baron (1980)				
	Other Pulmonary measures	NS	43 (2)	46 (2)
Lisano et al. (2019b)				
Maksud and Baron (1980)				
	Strength & endurance measures	NS	10 (1)	12 (1)
Lisano et al. (2019b)				
	Perceived Exertion	NS	43 (2)	46 (2)
Lisano et al. (2019b)				
Maksud and Baron (1980)				
	Resting Heart rate	^a^	58 (3)	61 (3)
Lisano et al. (2020)		^a^		
Lisano et al. (2019b)		NS		
Maksud and Baron (1980)		NS		
	Blood Pressure	NS	58 (3)	61 (3)
Lisano et al. (2020)				
Lisano et al. (2019b)				
Maksud and Baron (1980)				

Note: Resting Heart Rate is significantly higher in chronic cannabis users in Lisano et al. (2020) only

*NS* Not Significant, *PWC* Peak Work Capacity, *VO2Max* Maximal oxygen uptake

^a^ = Significant

### VO2Max and pulmonary measures

Three of the included studies measured participants’ performance on a treadmill with a breathing apparatus to determine VO2Max, while the remaining study (Maksud and Baron 1980) utilized a bicycle ergometer. The included studies did not find a significant difference in measures of VO2Max between groups, nor were the effects consistent in direction. Two of the included studies reported pulmonary measures, one using FEV1 (1 second forced expiratory volume), and the other using minute ventilation. Neither study reported a significant difference between cannabis users and non-users.

### PWC and other measures of strength and endurance

Lisano et al. (2019b) and Maksud and Baron (1980) measured PWC using a bicycle ergometer. Both studies found no significant difference between groups. Lisano, Smith et al. expressed that an effect may be found with a larger sample size, suggesting cannabis users may demonstrate less endurance in later stages of Wingate analysis. Only Lisano, Smith, et al. reported measures of strength and endurance other than PWC. These included grip strength, side plank times and joint torque. The differences between groups on these measures were not significant.

### Perceived exertion

Lisano et al. (2019b) reported on perceived exertion at termination of exercise which was not significantly different between groups. Maksud and Baron (1980) graphically reported perceived exertion throughout testing, finding only that the tobacco smoking group differed from other groups.

### Heart rate and blood pressure

Lisano et al. (2020) found a significant difference (*p* = 0.04) in resting HR between cannabis users and non-users, observing higher HR in the chronic cannabis users with a large effect size (*d* = .83). Two other studies found no significant difference in HR measurements between groups. Of three studies reporting BP outcomes, none found any significant difference between groups.

## Discussion

### Summary of results

Across the four included studies, no significant difference between cannabis users and non-users was observed for peak work capacity, cardiorespiratory fitness (VO2Max), other pulmonary measures (such as FEV1), strength and endurance measures (such as hand-grip strength), perceived exertion, or BP. Three studies reported resting HR, one of which reported a significantly higher resting HR among chronic cannabis users than non-users, opposing the findings of chronic cannabis use clinical trials (Benowitz and Jones 1975).

PWC and VO2Max are strongly correlated with physical fitness. The lack of difference in fitness measures in these results complement the results of Trinh et al. (2018), which reviewed the acute effects of cannabis consumption, finding limited and conflicting evidence of ergolytic or ergogenic effects on athletic performance. The present review addressed chronic cannabis use, revealing no significant impact of chronic use on exercise performance other than a difference in resting HR in one study. These findings suggest that present restrictions on cannabis use by sporting regulatory bodies are unjustified. While WADA’s stance of allowing CBD use and only banning THC (and by extension, whole cannabis products) in competition periods is helpful, access to highly refined CBD products is limited and may disadvantage less-wealthy athletes. WADA’s 150 ng/mL testing threshold for THC is generous compared to several US major league sporting bodies and the National Collegiate Athletic Association (NCAA), which recently increased their threshold from 15 ng/mL to 35 ng/mL in recognition of the realities of second-hand exposure (Burnsed 2019).

Acute cannabis consumption may reduce exercise pain and thus increase endurance (Huestis et al. 2011), but chronic use does not. In survey research, most people using cannabis with exercise reported reduced pain and/or increased their enjoyment of the exercise activity (Lisano et al. 2019a; YorkWilliams et al. 2019; Zeiger et al. 2019).

The included studies report body fat % and BMI between groups. Maksud and Baron (1980) found cannabis users to have significantly lower BMI and body fat %, a trend which is observed in most epidemiological studies, which Clark et al. (2018) suggests results from a metabolic effect caused by modulation of the endocannabinoid system.

This review found one study that directly related cannabis use to sport performance, however, it was excluded for using a post hoc design. Aedo-Muñoz et al. (2019) compared performance via video analysis between fighters that tested positive for cannabis during their bout and fighters that tested negative. The authors concluded that cannabinoids reduced fighter efficacy based on fewer head-strikes, however, a comparison of attempted strikes versus scored strikes reveals greater efficiency via success ratio. This emphasises the need for further research into performance in specific sports, rather than only physiological correlates of performance.

Two studies included in this review employed self-reported measures of physical activity levels, finding no significant differences between cannabis users and non-users, consistent with Ong et al.'s (2020) analysis of accelerometer detected physical activity, which determined no difference in light or moderate/vigorous physical activity between 249 cannabis users and 1843 non-users. Contrastingly, several longitudinal studies found significantly more cannabis users meeting WHO guidelines for physical activity than non-users (Korn et al. 2018; Ngueta et al. 2015; Rajavashisth et al. 2012).

### Limitations

As all studies revealed in this review were cross-sectional in design, causality cannot be established. As participants were asked to abstain from cannabis for 12 h prior to testing in Lisano et al. (2019b) and Lisano et al. (2020), and from smoking on the day of testing in Maksud and Baron (1980), withdrawal effects may confound the results. Wade et al. (2019b) required an abstinence period of 21 days so that withdrawal symptoms would subside and excluded participants for positive urinalysis, potentially deterring many cannabis users and likely affecting the representativeness of their sample. Lisano et al. (2019b, 2020) performed mass spectrometry on blood samples to verify cannabis use or non-use status. The remaining two studies relied entirely on self-reported cannabis use. Lisano et al. (2019a, 2020) specified that participants be physically active; Maksud and Baron used labourers for their sample, who are generally active in their job; Wade, Gilbart, et al. did not select participants based on fitness related factors. Three studies used a treadmill for VO2 measurement, while Maksud and Baron used a bicycle ergometer. PWC was also measured with two distinct protocols, Maksud and Baron used increasing work rate, while Lisano, Smith, et al. used the now prevalent Wingate methodology. The different protocols likely produce equally valid results, but complicate comparisons. Only Maksud and Baron, who conducted all testing in the morning, specified the time of day that fitness measures were conducted, thus diurnal variation effects may confound results. In Lisano, Smith, et al. dropouts occurred between stages of the study, potentially affecting the results of the strength and core endurance measures. Furthermore, all studies involved small samples that were not randomly sampled, and therefore may not be representative of the broader population.

### Future research considerations

Further research in the area of cannabis and exercise is needed as cannabis is increasingly legalised worldwide. Studies of the acute and chronic effects of cannabis on performance in specific sports, would be of value in addition to the literature on physiological measures. Cohort studies investigating differences in performance gain/loss over time between cannabis users and non-users are needed, as presently only cross-sectional studies are available. Comparison of chronic cannabis users and non-users in a crossover trial testing performance both with and without cannabis would aid in reducing potential confounds of withdrawal in chronic cannabis users.

## Conclusions

There have been few studies on the interactions between cannabis and exercise performance, and further research is urgently needed. Other than the acute effect of bronchodilation and consequent increased FEV1, there appears to be no reason based on current data to believe that cannabis has any significant ergogenic effect. In chronic use, there appears to be no ergolytic effect, however this is drawn exclusively from cross-sectional data. Increasingly, evidence supports the use of cannabis and cannabinoid products for pain, recovery, sleep, and appetite related applications and these substances are becoming recognised as valid medical interventions and alternatives to medications such as opioids and benzodiazepines. More data is needed on any auxiliary effects which may indirectly improve performance. The mental health and wellbeing of athletes is paramount, especially considering the psychological pressures faced, and cannabinoid therapies may provide ethical ways of supporting them without substantial ergogenic potential.

## Data Availability

Not applicable.

## Abbreviations

WADA: World Anti-Doping Agency
VO2Max: Maximal Oxygen Uptake
PWC: Peak Work Capacity
BP: Blood Pressure
HR: Heart Rate
FEV1: One-second Forced Expiratory Volume
THC: Tetrahydrocannabinol
CBD: Cannabidiol
eCB: Endocannabinoid
